# Supplementary material for: Hypersonic levitation and spinning: paving the way for enhanced single-cell analysis via contactless tissue dissociation
Source: Commun Eng. 2025 Sep 26;4:167. doi: 10.1038/s44172-025-00497-0 (PMC12475160; doi:10.1038/s44172-025-00497-0)
Supplement: Supplementary file 2 — Supplementary Information [file 44172_2025_497_MOESM2_ESM.pdf]

Supplementary Materials for

Hypersonic Levitation and Spinning: Paving the Way for Enhanced Single-Cell Analysis via Contactless Tissue Dissociation

Yang Bai *et al.*
Corresponding author: Xuexin Duan, xduan@tju.edu.cn

The PDF file includes:

Figure S1 to S8
Table S1
Legends for movies S1

Other Supplementary Material for this manuscript includes the following:

Movies S1

Table S1

Table S1 Flow cytometry probe panels.

Assay	Antibody		Fluorophore	Positive cells
	Clone	Dilution(μg/mL)		
CA9	053	4	PE	Renal cancer cells
CD45	HI30	2.5	FITC	Leukocytes
Viability	N/A	2.5	7-AAD	Dead cells

Table S1. Flow cytometry probe panels.

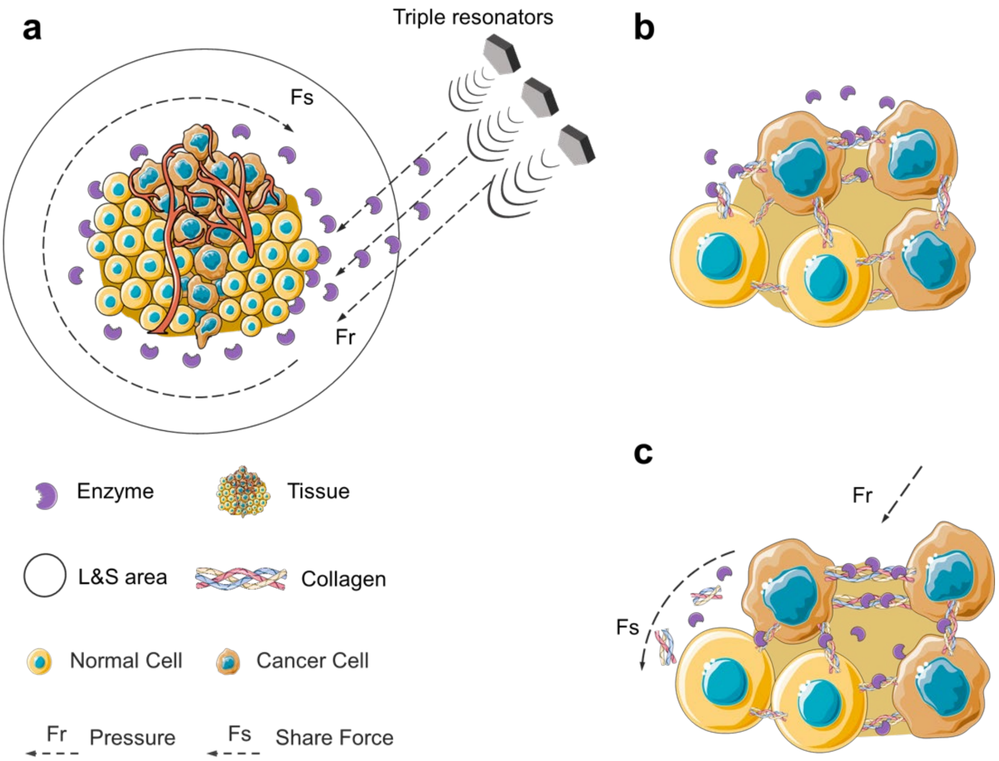

Figure S1. Mechanism of tissue dissociation using the HLS method. (a) Driven by the triple-resonator probe, eccentric pressure (*Fr*) and shear force (*Fs*) are generated, which, in combination with the conical structure, create a levitation and spinning area. In this area, the tissue block remains stably levitated and self-rotating. (b) Without the HLS method, enzymes can only bind to the collagen fibers on the tissue surface, failing to penetrate deeper layers of the tissue. Additionally, enzymatic digestion products are not easily removed. (c) Using the HLS method, the impact of *Fr* widens the gaps between tissues, allowing enzymes to bind to deep collagen for digestion. *Fs* strips the enzymatic digestion products, exposing underlying sites for further enzymatic binding.

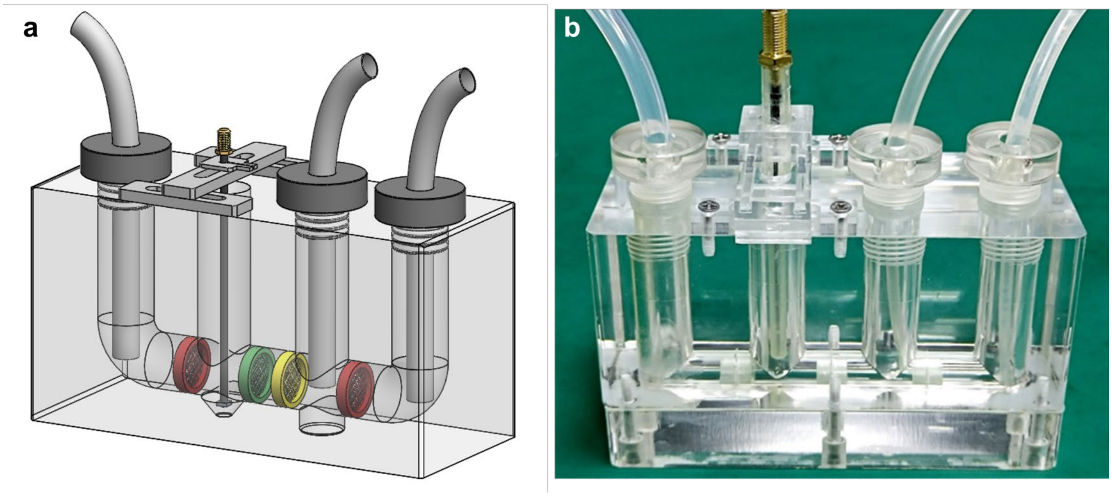

Figure S2. The automated tissue dissociation device. (a) Structural diagram of the automated tissue dissociation device. (b) Physical image of the automated tissue dissociation device.

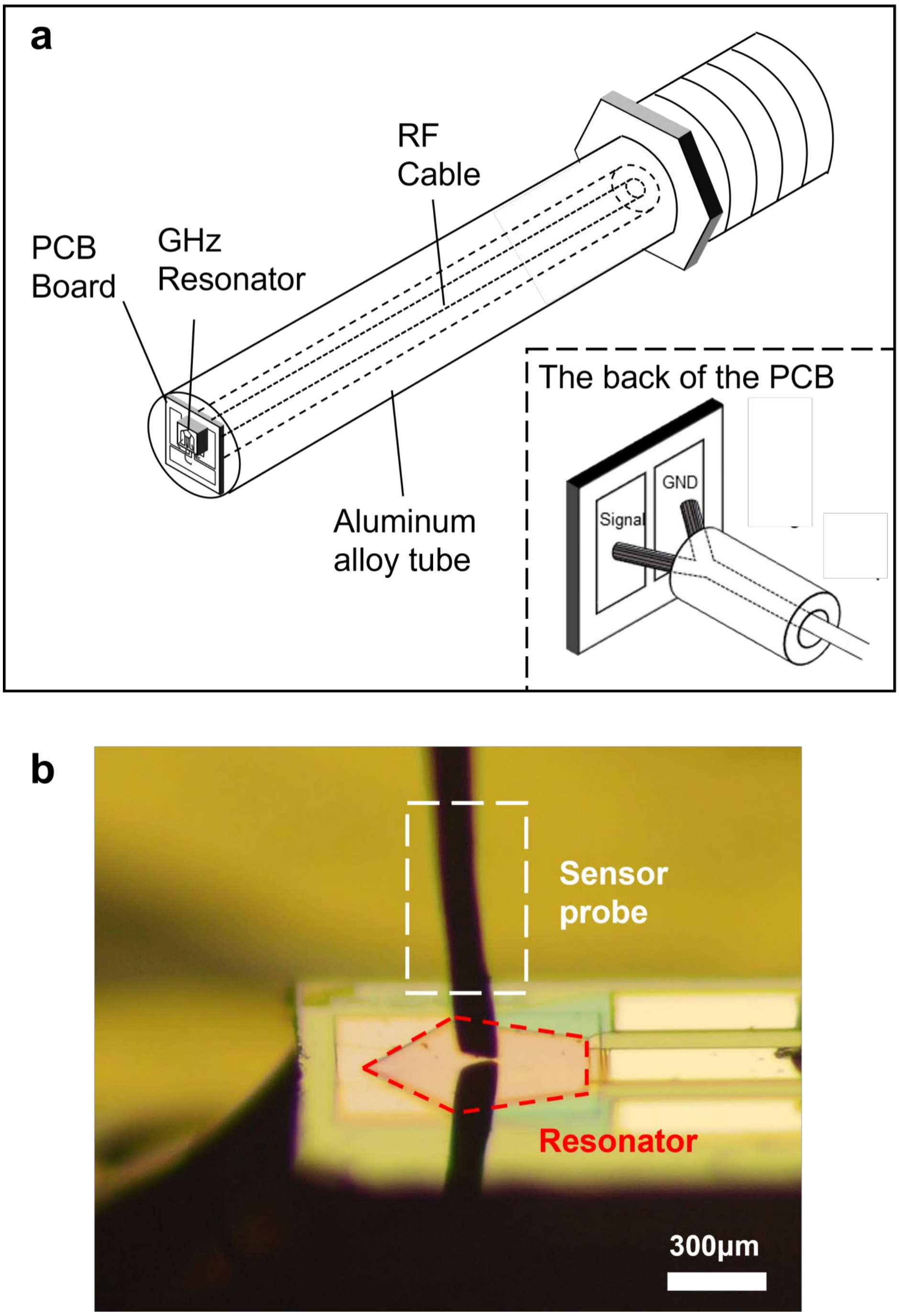

Figure S3. Schematic and measurement setup of the acoustic probe system. (a) Schematic illustration of the probe assembly structure. The RF wire splits into a signal line and a ground line at the top, which are soldered to two pads on the back side of the PCB. The GHz resonator is mounted on the front side of the PCB using AB adhesive and electrically connected via three gold wires (ground–signal–ground, from left to right). The entire assembly is enclosed in an aluminum alloy casing to protect the internal components. (b) Experimental setup for measuring the jetting force generated by the resonator using a force sensor. The sensor probe is positioned 100 μm above the resonator surface.

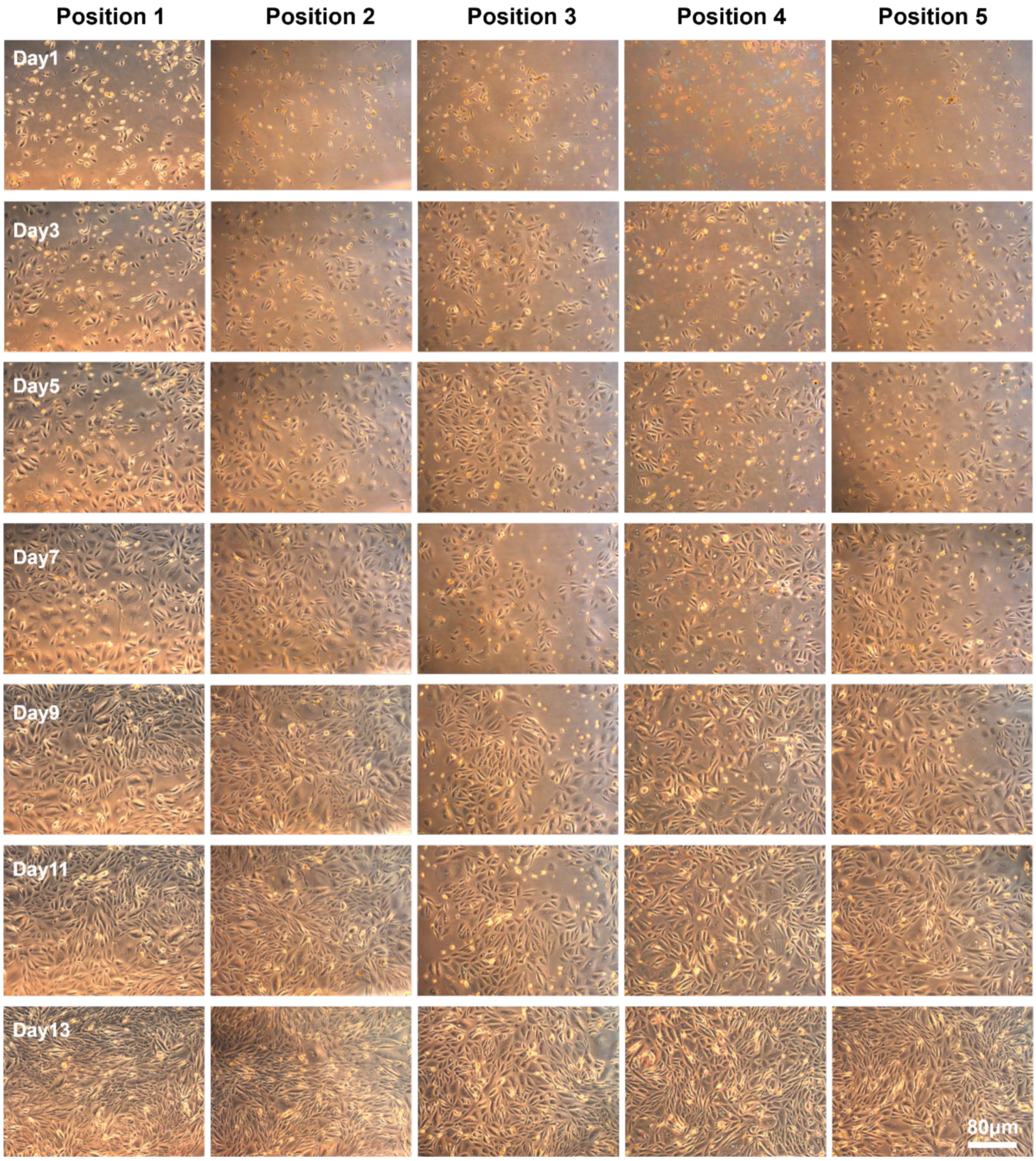

Figure S4. Images of cell growth in the HLS device group during 13 days of primary culture, with tracking at five fixed positions.

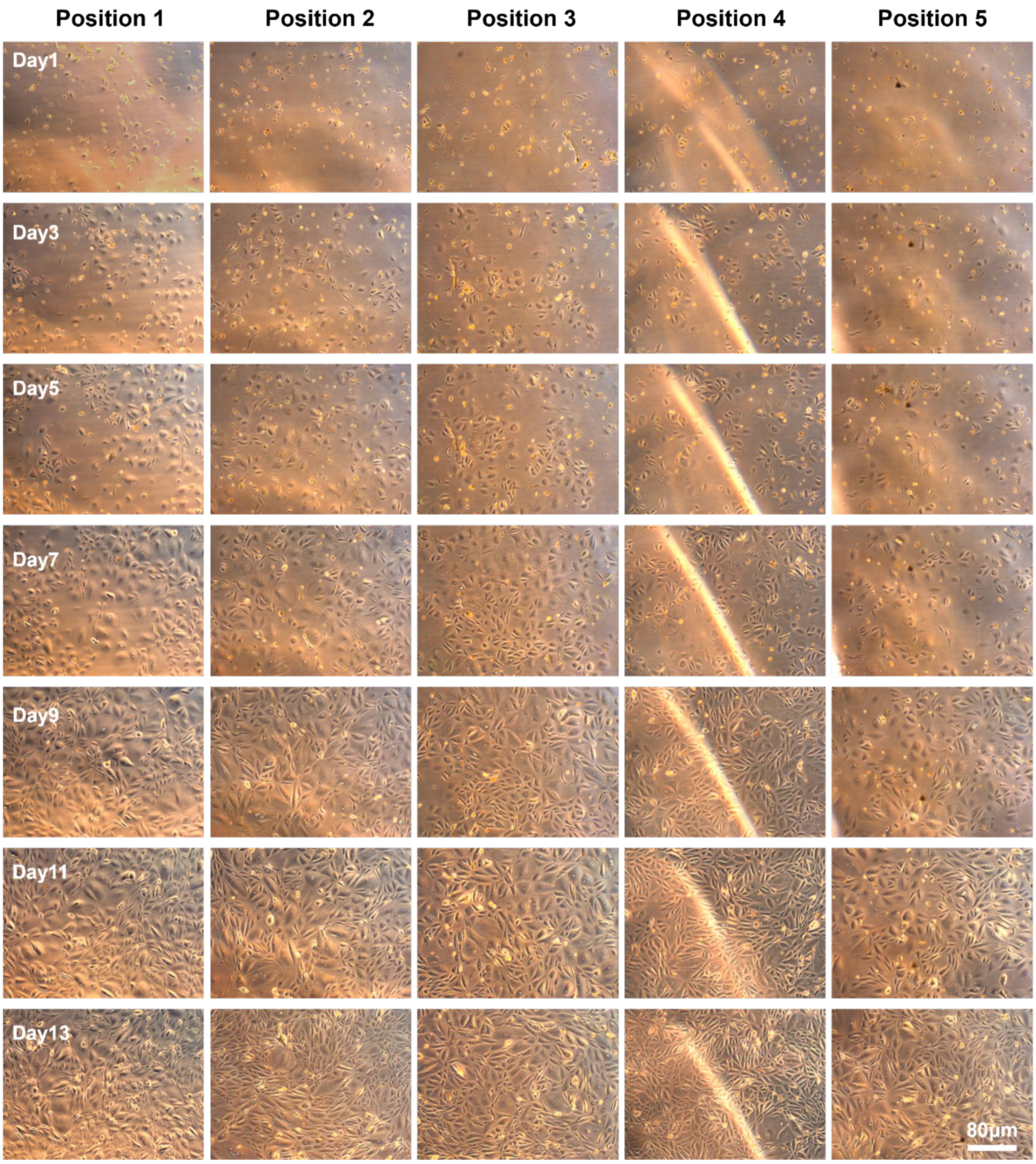

Figure S5. Images of cell growth in the shaker group during 13 days of primary culture, with tracking at five fixed positions.

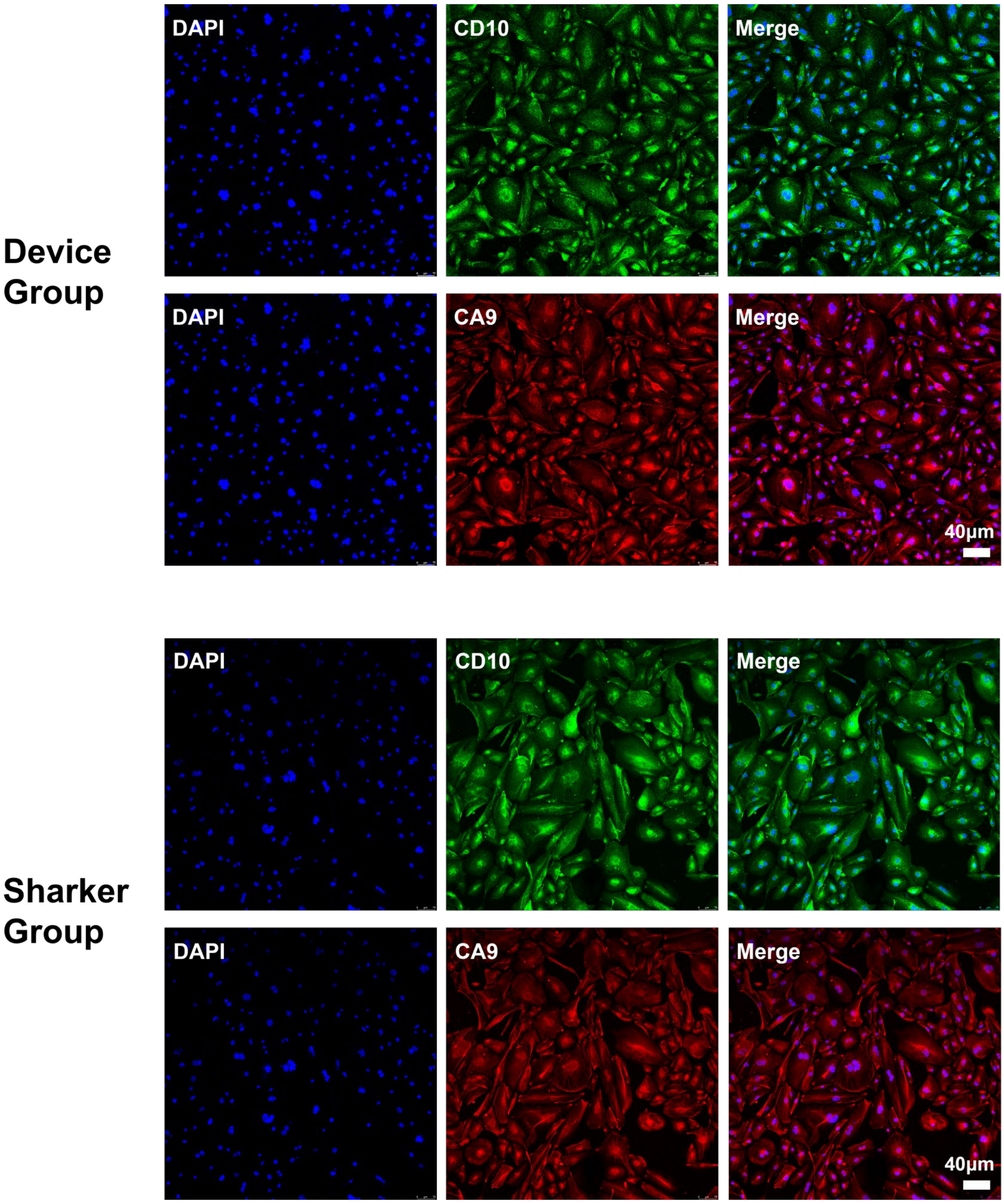

Figure S6. Immunofluorescence characterization of human renal cancer tissues dissociated by the device group and the shaker group respectively revealed typical features of clear cell renal carcinoma ($CA9^{+}$, $CD10^{+}$).

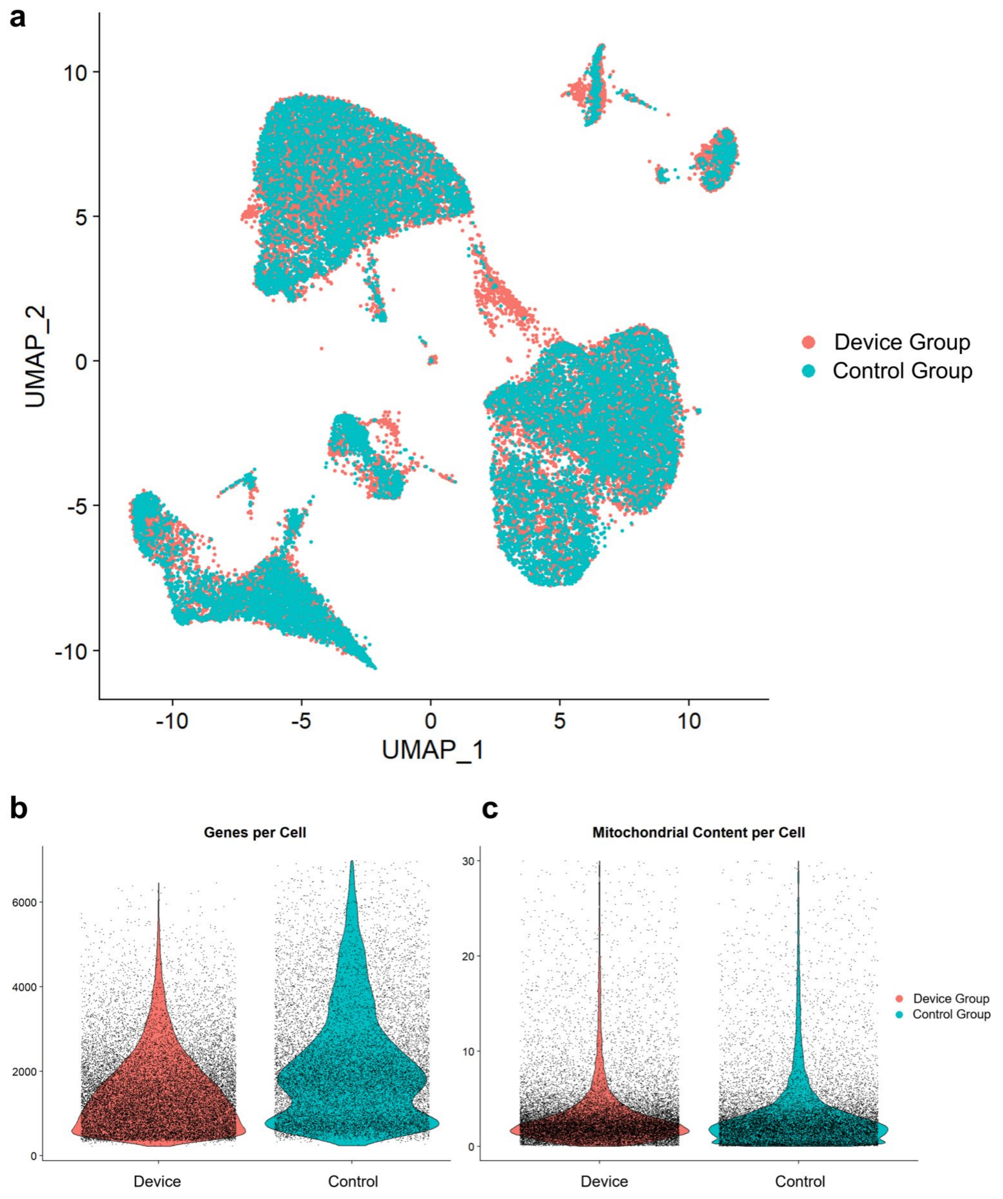

Figure S7. Side-by-side comparison of UMAP visualizations and key metrics derived from single-cell RNA sequencing data obtained using the traditional dissociation method and the HLS method. (a) Differences in cell clustering UMAP plots between the device group using the HLS method and the control group using the traditional dissociation method. (b) Comparison of gene counts per cell. (c) Comparison of mitochondrial gene content per cell.

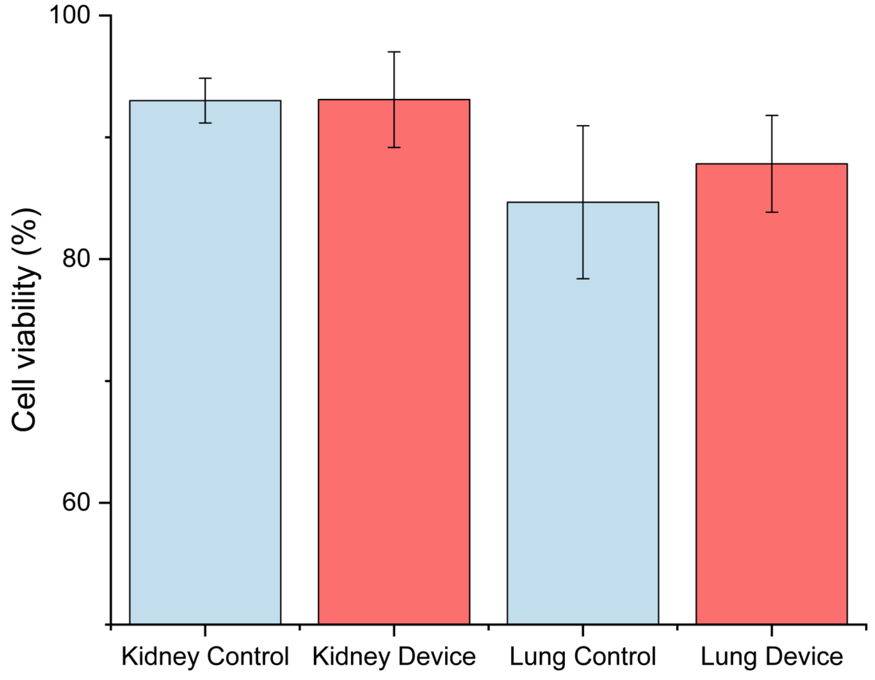

Figure S8. Comparison of cell viability after dissociation of rat kidney and lung tissues using the traditional shaker method (1 hour) and the HLS device (15 minutes).
