# Supplementary material for: Hypersonic levitation and spinning: paving the way for enhanced single-cell analysis via contactless tissue dissociation
Source: Commun Eng. 2025 Sep 26;4:167. doi: 10.1038/s44172-025-00497-0 (PMC12475160; doi:10.1038/s44172-025-00497-0)
Supplement: Supplementary file 3 — Description of Additional Supplementary Files [file 44172_2025_497_MOESM3_ESM.pdf]

Description of Additional Supplementary Files

File name- Supplementary Movie S1

File description – Characterization of Power and Rotational Speed in Hypersonic Streaming Levitation and Spinning
